# Association between school environment with sedentary behavior and physical activity intensity in children

**DOI:** 10.1038/s41598-023-33732-9

**Published:** 2023-04-28

**Authors:** Diego Sales, João Pedro da Silva Junior, Raiany Rosa Bergamo, Luis Carlos de Oliveira, Gerson Ferrari, Victor Matsudo

**Affiliations:** 1 Study Center of the Physical Fitness Laboratory of São Caetano Do Sul (CELAFISCS), Rua Santo Antonio, 50 – 5º andar - salas 504/505 - Centro, São Caetano Do Sul, SP Brazil; 2 Escuela de Ciencias de La Actividad Física, el Deporte Y La Salud, Universidad de Santiago de Chile, Santiago, Chile; 3 Facultad de Ciencias de La Salud, Universidad Autónoma de Chile, Providencia, Chile

**Keywords:** Health care, Risk factors

## Abstract

We examined the association between indicators of the school environment with sedentary behavior and different intensities of physical activity in children. The study that included 515 children (265 boys) aged 9–11 years old from public and private schools in the city of São Caetano do Sul. Sedentary behavior and different intensities of physical activity were evaluated with an accelerometer. Inside school environment (policies, supervision committee, extracurricular activities, breaks, and access to school facilities) was evaluated using a questionnaire. Policies and practice (β: 8.49; 95% CI: 3.62–13.36), supervision committee (5.42; 0.64–10.19), inter-school competitions (2.40, 2.25–2.55), breaks of 15–29 min/day (6.87; 2.20–10.75), and outdoor sports field (5.40; 0.37–10.44), were positively associated with moderate-to-vigorous-intensity physical activity. Furthermore, crossing guards (7.65; 3.00–12.30) were positively associated with moderate-to-vigorous-intensity physical activity. We concluded that an association was found between school environment indicators with higher levels of physical activity and greater odds of meeting physical activity guidelines.

## Introduction

Previous studies have shown that physical activity is associated with indicators in body composition, physical fitness, cardiometabolic biomarkers, academic performance, and psychological well-being in children and adolescents^1–4^. Despite these benefits, fewer than 20% of adolescents worldwide and only 29% of Brazilian children aged between 11 and 17 meet the international recommendation of physical activity^5^. For children and teenagers from 5 to 17 years, the 2020 World Health Organization Guidelines recommends an average of 60 min/day of moderate-to-vigorous physical activity^5^.

Physical activity is determined not solely by behavior and choices of individuals, but also by sociocultural and environmental factors, such as school environments^6,7^. Factors that can affect children’s physical activity during school hours include physical, social, or institutional structures. These factors are in line with ecological models of change in behavior in which an environment can influence lifestyle behaviors life^8^. In addition, monetary aspects can affect the schools’ ability of offerring opportunities to encourage the practice of physical activity among children in the school environment. There is a growing body of research into the influence of the school environment, particularly barriers and facilitators, in children physical activity^8,9^. Two systematic reviews^8,9^ demonstrated that schools can improve children’s physical activity when they emphasize the school environment, availability of physical extracurricular activities and the provision of resources for physical activity during the school day, thus create a “culture” of physical activity.

Existing evidence in school environments and different intensities of objectively measured physical activity, such as accelerometry, especially in children from high-income countries has been reported in the literature^10,11^. On the other hand, studies on this topic in low- to middle-income countries, such as Brazil is scarce^12^. In addition, studies that use objective measures of physical activities in children such accelerometry are necessary to investigate the indicators of the school environment associated with different intensities of physical activity such as (e.g., sedentary behavior, light-intensity and moderate-to-vigorous-intensity) more comprehensively. Therefore, the present study was to analyze the association between the school environment with sedentary behavior and different physical activity intensities assessed with accelerometry in children.

## Methods

### Study of childhood obesity, lifestyle and environment (ISCOLE)

This study is part of the International Study of Childhood Obesity, Lifestyle, and the Environment (ISCOLE) is characterized as a multicenter cross-sectional study involving 12 countries from five main geographic regions of the world. The main objective of ISCOLE was to investigate the relationships between environmental, social and political factors with lifestyle (diet and activity physical) and obesity of children aged 9–11 years^13^. The present study focuses on the data collected in the municipality of São Caetano do Sul, located in the state of São Paulo.

There is variability in socioeconomic status between schools in the region of São Caetano do Sul. Public schools represent the lower and lower-middle socioeconomic strata, while private schools reflect the middle and upper-middle class. Random lists of public and private elementary schools in the region were generated, and schools were selected from each list at a ratio of 4 (public) to 1 (private). If a school refused to participate in the study, it was replaced by the next school on the list. Twenty schools were sampled (16 public and 4 private) to generate a sample of 25 to 30 children from each school. Given that the primary sampling strategy was based on schools, ISCOLE data collection was conducted during the school year with the aim of obtaining 500 children (approximately 50% of each sex) aged 9–11 years, as per the sample calculation^13^. All children attending fifth grade (9–11 years old) were invited to participate in the study^14,15^.

Data collection was performed between March 2012 and April 2013, and all assessments were performed for one full week per school. In total, 584 children (277 boys) met the respective inclusion criteria: aged between 9 and 11 years; being enrolled in the São Caetano do Sul school system; and without clinical or functional limitations. The final sample of the present study was consisted of 515 children (265 boys). ISCOLE details have been published previously^13–15^.

### Physical activity assessment

To objectively measure sedentary behavior and the associated physical activity intensities (light, moderate, vigorous, and moderate-to-vigorous), we used the Actigraph GT3X accelerometer (ActiGraph, Ft. Walton Beach, United States United). The instrument was used at the waist on an elastic belt, on the right midaxillary line and children were encouraged to use the accelerometer 24 h a day for at least seven days, including two weekend days. Children were instructed to remove the accelerometer only for activities water (e.g., bathing and swimming). To increase adherence, the study team instructed the children on how to use the accelerometer during the assessment start at school. During the week of use, participants were contacted by phone to make sure they were using the equipment and to clarify any doubts.

The minimum amount of accelerometer data that was considered acceptable for analysis was four days (including at least one weekend day), with at least 10 h per day of wear time, after removal at the sleep time^16,17^. Data were collected at a sampling rate of 80 Hz. Raw accelerometer data were integrated into 1 s epochs and later aggregated to 60 and 15 s epochs with the low-frequency extension filter enabled^18^. An automated algorithm was used to detect children's sleep-period and non-wear time (any sequence of at least 20 consecutive minutes of zero activity counts)^19,20^. Once nocturnal sleep episode time and non-wear time were computed, waking wear time and the dissimilar activity levels and sedentary behvaior were calculated and identified using the 15 s epoch data. We used the cutoff point of ≤ 25 counts/15 s for the behavior total sedentary, 26–573 counts/15 s for light physical activity, 574–1002 counts/15 s for moderate physical activity, and ≥ 1003 counts/15 s for vigorous physical activity. In addition, we also consider ≥ 574 counts/15 s for moderate-to-vigorous physical activity^18^. The cut-off points used capture the sporadic nature of children’s activities and provide classification accuracy between current cut-off points available for total sedentary behavior and physical activity in children^17^. In the analyses, we also categorized children into achieved (mean ≥ 60 min/day) and not achieved (mean < 60 min/day) current recommendations for moderate-to-vigorous physical activity according to the World Health Organization^21^. Sedentary behavior and physical activity intensities were defined only during school using school-day schedules provided by each participating school. The full accelerometer protocol has been published elsewhere^22^.

### School environment assessment

The school environment was assessed using the ISCOLE School Environment Questionnaire^13^ which was completed by a school administrator during the visit to school. The questionnaire was also used in previous studies^14,23,24^. The school administrator questionnaire was adapted from the healthy eating and physical activity modules of the Healthy School Planner^25^ used in the Canadian School Health Action, Planning and Evaluation System (SHAPES)^26^. In this study, we investigated only inside school environment indicator separately.

For inside school was considered the follows indicators: the school have written policies or practices concerning physical activity, committee that oversees or provides guidance on policies and practices related to physical activity and healthy eating, inter-school competitions, sports schools, academic hobby activities, arts schools, breaks of 15–29 min/day, gymnasium, other large room suitable for physical activity, running track, outdoor sports field, art room, indoor swimming pool, music room, playground, and playground equipment. The response categories were altered in an attempt to reduce potential subjectivity. We used the questions and responses categories presented in Table 1.

**Table 1:** Indicators of the school environment.

Variable	Responses	Analyzed Category
Does your school have written policies or practices concerning physical activity	No; Yes, existing written policies; Yes, written policies still under development; Yes, practices	No; Yes
Committee that oversees or provides guidance of policies and practices concerning physical activity and healthy eating	No; Yes, for both; Yes, only for physical activity; Yes, only for healthy eating	No; Yes
Inter-school competitions; sports schools (including dance); academic hobby activities (e.g., chess and draughts); arts schools (e.g., theater, music, and photography)	Not available; < 10%; 10–24%; 25–49%; ≥ 50%	No; Yes
Breaks of 15–29 min/day	0; 1; 2; 3	No; Yes
Access to school facilities: gymnasium; other rooms for physical activity, fitness, or aerobic room; running track; outdoor sports; art rooms; swimming pool; music rooms; playground; playground equipment	Yes, on grounds only; Yes, off grounds only; Yes, both on and off grounds; No; Don't know	No; Yes

### Sociodemographic variables

A parent or legal guardian was invited to complete the Demographic and Family Health Questionnaire^13^ related to sex, ethnicity (white, black, brown, and others), and number of siblings of the child. In the family context, we use the income annual family income (BRL) as an indicator of socioeconomic level and was categorised as quartile (≤ BRL 19.620; BRL 19.621 to BRL 32.700; BRL 32.701 to BRL 58.860, and ≥ BRL 58.861), being the first quartile the poorest group and the fourth quartile the wealthiest group. In addition, the type of school (public or private) was used as an indicator of level socioeconomic status from lower to higher socioeconomic status.

### Statistical analysis

Descriptive analysis was presented as mean and standard deviation for variables continuous variables and the frequency and percentage for the categorical variables. The test of Kolmogorov–Smirnov was used to verify data distribution. Differences between sexes were analyzed using Student’s t test for the independent variables of the samples (continuous variables) and the chi-square test (variables categorical).

Linear regression (β; 95% confidence interval [95%CI]) was used to evaluate the association of school environment indicators with behavior sedentary and the different intensities of physical activity (light-intensity, moderate-intensity, and vigorous-intensity). Binary logistic regression (odds ratio [OR] and 95%CI) was used to analyze the association between the school environment and achieved (mean ≥ 60 min/day) or not achieved (mean < 60 min/day) of the international recommendations of physical activity of physical activity as suggested by the World Health Organization^21^. Both regression models were multilevel, including school as an effect random, adjusted for sex, ethnicity, school type, number of siblings, family income year total, and school. The significance level adopted was *p *< 0.05. Analyses were carried out using SPSS, version 24 (SPSS Inc., IBM Corp., Armonk, New York, NY, USA).

### Ethics approval

The Institutional Review Board of the Pennington Biomedical Research Center in Baton Rouge, USA (coordinating center) has approved the ISCOLE protocols. In addition, the Research Ethics Committee of the Federal University of São Paulo (number: 332.529) also approved the local protocol. Written informed consent was obtained from parents or legal guardians, and child consent was also obtained as required by local Ethics Review Boards prior to study participation. The study was performed in accordance with relevant guidelines and regulations.

## Results

The mean values presented in Table 2 are significant for age, annual family income, sedentary behavior and for the different intensities of physical activity, except for light-intensity physical activity. Overall, girls were older than boys, had higher family income and greater sedentary behavior. Boys, on the other hand, showed greater involvement in different intensities of physical activities.

**Table 2:** Descriptive analysis (mean [SD] or n [%]) of socio-demographic and accelerometry variables.

Variable	Total (n = 515)	Boys (n = 265)	Girls (n = 297)	*p*- value
Age (Years)	10.07 (0.52)	9.78 (0.41)	10.34 (0.47)	< 0.001
Ethnicity (%)*				0.658
White	383 (74.4)	203 (76.7)	180 (72.0)	
Black	38 (7.4)	17 (6.4)	21 (8.4)	
Brown	72 (14.0)	35 (13.2)	37 (14.8)	
Other	22 (4.3)	10 (3.8)	12 (4.8)	
Annual family income (BRL)**, (%)				< 0.001
≤ BRL 19.620	193 (37.5)	95 (35.8)	116 (39.2)	
BRL 19.621 to R$ 32.700	134 (26.0)	78 (29.5)	67 (22.6)	
BRL 32.701 to BRL 58.860	113 (21.9)	58 (22.1)	65 (21.6)	
≥ BRL 58.861	75 (14.6)	34 (12.6)	49 (16.6)	
Accelerometry (min/day)***				
Sedentary behavior	200.30 (27.50)	196.8 (28.1)	202.9 (26.71)	< 0.001
Light-intensity physical activity	134.91 (52.72)	136.0 (20.76)	133.86 (21.41)	0.260
Moderate-intensity physical activity	16.58 (6.59)	19.50 (6.80)	14.08 (5.14)	< 0.001
Vigorous-intensity physical activity	7.02 (4.48)	8.98 (5.01)	5.20 (2.58)	< 0.001
Moderate-to- vigorous physical activity	23.80 (10.56)	28.49 (11.20)	19.29 (7.42)	< 0.001

*SD* standard deviation.

*(%) percentage.

**BRL Brazilian real.

***min/day minutes per day.

Table 3 presents the description of the school environment in which most schools were public (80.0%). Regarding the inside school, a large proportion had written policies and practices on counseling for physical activity and healthy eating (90.2%), offered extracurricular activities such as interscholastic competitions (85.0%), sports (80.5%), hobby academic activities (71.6%), art schools (69.5%). In addition, they offered access to the following facilities: gymnasium (79.2%), art room (75.0%), playground (61.8%), and playground equipment (58.3%).

**Table 3:** Descriptive analysis (%) of school environment.

Variables	%
Type of school	
Public/Private	80.0/20.0
Does your school have written policies or practices concerning physical activity	
No/Yes	9.8/90.2
Committee that oversees or provides guidance on policies and practices related to physical activity and healthy eating	
No/Yes	52.4/45.6
Inter-school competitions	
No/Yes	15.0/85.0
Sports schools	
No/Yes	12.5/80.5
Academic hobby activities	
No/Yes	28.4 /71.6
Arts schools	
No/Yes	30.4/69.5
Breaks of 15–29 min/day	
No/Yes	75.0/25.0
Gymnasium	
No/Yes	20.8/79.2
Other large room suitable for physical activity	
No/Yes	41.7/58.3
Running track	
No/Yes	85.7/14.3
Outdoor sports field	
No/Yes	39.1/60.8
Art room	
No/Yes	25.0/75.0
Indoor swimming pool	
No/Yes	85.7/14.3
Music room	
No/Yes	50.0/50.0
Playground	
No/Yes	38.2/61.8
Playground equipment	
No/Yes	41.7/58.3

(%) percentage.

In the multilevel linear regression model, we found positive associations between schools indicators, such as physical activity policies and practices with vigorous-intensity (β: 4.91; 95% CI: 1.44; 8.38) and moderate-to-vigorous-intensity (β: 8.49; 95% CI: 3.62; 13.36). In addition, was found associations between extracurricular activities analyzed by inter-school competitions with moderate-intensity (β: 2.10, 95% CI: 1.60; 2.60), vigorous-intensity (β: 2.49, 95% CI: 1.06; 3. 92) and moderate-to-vigorous-intensity (β: 2.40, 95% CI: 2.25; 2.55); sports schools with vigorous-intensity (β: 3.70, 95% CI: 1.27; 5.93); arts schools with light-intensity (β: 6.37, 95% CI: 3.51; 9.23); breaks of 15–29 min/day were associated with light-intensity (β: 14.17; 95% CI: 4.54; 23.81), vigorous-intensity (β: 3.69; 95% CI: 1.65; 5.73) and moderate-to-vigorous-intensity (β: 6.87; 95% CI: 2.20; 10.75); the presence of a running track was associated with vigorous-intensity (β: 2.80; 95% CI: 1.61; 3.99) and indoor swimming pool with light-intensity (β: 10.80; 95% CI: 2.90; 18.70) (Table 4).

**Table 4:** Multilevel linear regression between school environment with sedentary behavior and physical activity intensities.

Variables	SB*	LPA**	MPA**	VPA**	MVPA**
	β (IC 95%) ***	β (IC 95%)	β (IC 95%)	β (IC 95%)	β (IC 95%)
Policies and practices					
No	Ref.	**Ref.**	Ref.	**Ref.**	**Ref.**
Yes	− 22.48 (− 48.98; 4.01)	**20.16 (0.02; 40.30)**	4.58 (− 1.74; 10.91)	**4.91 (1.44; 8.38)**	**8.49 (3.62; 13.36**)
Committee that oversees or provides guidance on policies and practices related to physical activity and healthy eating					
No	Ref.	Ref.	Ref.	Ref.	**Ref.**
Yes	− 7.21 (− 19.76; 5.30)	3.72 (− 5.83; 13.28)	8.49 (− 1.62; 18.61)	1.98 (− 0.07; 4.0)	5.42 (0.64; 10.19)
Inter-school competitions					
No	Ref.	Ref.	**Ref.**	**Ref.**	**Ref.**
Yes	2.88 (− 19.01; 24.78)	− 5.78 (− 22.56; 10.98)	**2.10 (1.60; 2.60)**	**2.49 (1.06; 3.92)**	**2.40 (2.25; 2.55**)
Sports schools					
No	Ref.	Ref.	Ref.	**Ref.**	Ref.
Yes	12.50 (− 3.29; 28.29)	− 1.28 (− 13.41; 10.84)	− 5.97 (− 9.69;−2.24)	**3.70 (1.27; 5.93)**	− 6.67 (− 12.61; − 0.73)
Academic hobby activities					
No	Ref.	Ref.	Ref.	Ref.	Ref.
Yes	− 3.45 (− 19.41; 12.50)	− 0.64 (− 12.88; 11.59)	0.13 (− 3.65; 3.93)	− 0.71 (− 3.30; 1.87)	− 0.58 (− 6.60; 5.44)
Arts schools					
No	Ref.	**Ref.**	Ref.	Ref.	Ref.
Yes	− 10.17 (− 24.34; 3.98)	**6.37 (3.51; 9.23)**	− 2.16 (− 5.53; 1.20)	1.36 (− 0.93; 3.67)	− 0.79 (− 6.14; 4.56)
Breaks 15–29 min/day					
No	Ref.	**Ref.**	**Ref.**	**Ref.**	**Ref.**
Yes	− 5.49 (− 18.15; 7.16)	**14.17 (4.54; 23.81)**	3.28 (0.26; 6.31)	**3.69 (1.65; 5.73)**	**6.87 (2.20; 10.75)**
Gymnasium					
No	Ref.	Ref.	Ref.	Ref.	Ref.
Yes	− 1.81 (− 16.34; 12.71)	6.37 (− 4.74; 17.50)	2.32 (− 1.15; 5.80)	− 0.25 (− 2.62; 2.11)	2.06 (− 3.45; 7.58)
Other large room suitable for physical activity					
No	Ref.	Ref.	Ref.	Ref.	Ref.
Yes	1.12 (− 11.65; 13.91)	− 1.08 (− 10.88; 8.71)	− 2.55 (− 5.61; 0.49)	− 0.47 (− 2.55; 1.61)	− 3.02 (− 7.88; 1.82)
Running track					
No	Ref.	Ref.	Ref.	**Ref.**	Ref.
Yes	− 8.71 (− 35.47; 18.04)	16.68 (− 3.78; 37.15)	0.65 (− 5.76; 7.08)	**2.80 (1.61, 3.99)**	0.30 (− 9.86;10.48)
Outdoor sports field					
No	Ref.	Ref.	**Ref.**	Ref.	**Ref.**
Yes	1.81 (− 11.49; 15.11)	− 6.12 (− 16.30; 4.06)	3.44 (0.26; 6.62)	1.96 (− 0.19; 4.12)	5.40 (0.37; 10.44)
Art room					
No	Ref.	Ref.	Ref.	Ref.	Ref.
Yes	9.90 (− 5.10; 24.91)	− 4.00 (− 15.52; 7.51)	1.20 (− 2.40; 4.0)	0.92 (− 1.52; 3.37)	2.12 (− 3.58; 7.83)
Indoor swimming pool					
No	Ref.	**Ref. **	Ref.	Ref.	Ref.
Yes	− 19.61 (− 71.07; 31.85)	**10.80 (2.90; 18.70)**	− 10.09 (− 27.36; 7.81)	− 5.19 (− 13.57; 3.18)	− 20.28 (− 39.76; − 0.80)
Music room					
No	Ref.	Ref.	Ref.	Ref.	Ref.
Yes	− 0.15 (− 12.56; 12.25)	2.07 (− 7.44; 11.58)	− 0.71 (− 3.68; 2.26)	0.82 (− 1.20; 2.84)	0.10 (− 4.60; 4.82)
Playground					
No	Ref.	Ref.	Ref.	Ref.	Ref.
Yes	9.29 (− 4.28; 22.87)	1.08 (− 9.27; 11.43)	0.94 (− 2.29; 4.19)	0.45 (− 1.78; 2.68)	1.39 (− 3.79; 6.59)
Playground equipment					
No	Ref.	Ref.	Ref.	Ref.	Ref.
Yes	11.59 (− 1.36; 24.55)	− 2.06 (− 12.02; 7.90)	− 0.48 (− 3.60; 2.62)	− 0.06 (− 2.18; 2.05)	− 0.55 (− 5.49; 4.38)

Values in bold indicate significant associations.

*SB sedentary behavior.

**Intensities of physical activity: *LPA* light physical activity, *MPA* moderate physical activity, *VPA* vigorous physical activity, *MVPA* moderate-vigorous physical activity.

***95% *CI* confidence interval. Multilevel regression, including school as a random effect, adjusted for sex, ethnicity, school type, number of siblings, total annual household income, and school.

In the multilevel logistic regression model, it can be seen that variables inside the school, such as presence of a committee that oversees or provides guidance on policies and practices related to physical activity and healthy eating (OR: 2.42; 95% CI: 1.98; 2.86), extracurricular activities, such as inter-school competitions (OR: 1. 94; 95% CI: 1.02; 3.69) and sports schools (OR: 1.59; 95% CI: 1.01; 2.19), have intervals of 15–29 min/day (OR: 1.51; 95% CI: 1.05; 2.19), and access to outdoor sports (OR: 1.90; 95% CI: 1.50; 2.30) were associated with higher odds of being met with physical activity recommendations (Table 5).

**Table 5:** Multilevel logistic regression (OR [95%CI]) of school environment on achievement of physical activity recommendation of children.

Variables	OR*	95% CI**	*p-* value
Policies and practices			
No	Ref.		
Yes	1.98	0.85; 4.60	0.110
Committee that oversees or provides guidance on policies and practices related to physical activity and healthy eating			
No	Ref.		
Yes	**2.42**	**1.98; 2.86**	**0.031**
Inter-school competitions			
No	Ref.		
Yes	**1.94**	**1.02; 3.69**	**0.040**
Sports schools			
No	Ref.		
Yes	**1.59**	**1.01; 2.51**	**0.043**
Academic hobby activities			
No	Ref.		
Yes	1.07	0.68; 1.70	0.744
Arts schools			
No	Ref.		
Yes	1.01	0.67; 1.52	0.957
Breaks of 15–29 min/day			
No	Ref.		
Yes	**1.51**	**1.05; 2.19**	**0.021**
Gymnasium			
No	Ref.		
Yes	1.17	0.76; 1.80	0.456
Other large room suitable for physical activity			
No	Ref.		
Yes	1.43	0.98; 2.07	0.059
Running track			
No	Ref.		
Yes	1.35	0.61; 2.90	0.466
Outdoor sports field			
No	Ref.		
Yes	**1.90**	**1.50; 2.30**	**0.031**
Art room			
No	Ref.		
Yes	1.16	0.75; 1.80	0.496
Indoor swimming pool			
No	Ref.		
Yes	5.00	0.59; 41.90	0.137
Music room			
No	Ref.		
Yes	1.10	0.76; 1.57	0.606
Playground			
No	Ref.		
Yes	1.09	0.73; 1.61	0.664
Playground equipment			
No	Ref.		
Yes	1.29	0.88; 1.88	0.182

Values in bold indicate significant associations.

Multilevel logistic regression, including school as a random effect, adjusted for sex, ethnicity, school type, number of siblings, total annual household income and school.

*OR odds ratio.

**95% *CI* confidence interval.

## Discussion

This paper analyzed the association between school environment indicators with sedentary behavior and the different intensities of physical activity in children. Overall, we found distinct associations between inside indicators of the school environment with the different intensities of physical activity. For instance, we found positive associations between policies and practices, a committee that oversees or provides guidance on policies and practices related to physical activity and healthy eating with increasing activity times vigorous-intensity and moderate-to-vigorous-intensity physical activity.

Corroborating previous findings^27^, in which there was a greater percentage (78.6%) of students who were more active when they knew the message of the Agita São Paulo program after its intervention as a political public school to increase the physical activity levels of public-school student’s state education in the state of São Paulo. Another recent study^28^, reported increases in active transport when schools had policies for promotion of active transport, highlighting the importance of the federal spheres, state, and municipal authorities to propose more policies like others already established^29–31^ and resources to promote physical activity as well as the translation of these policies into practices by schools^32,33^ with the aim of increasing levels of physical activity of children and adolescents. There were differences in the magnitude of associations between within-school variables related to extracurricular activities. These differences are possibly due to differences in energy expenditure between these practices.

We found positive associations between the break and increased levels of physical activity intensities and higher odds of students meet the recommendations physical activity, corroborating other findings^34,35^, but Costa and collaborators^36^ showed that after an intervention in the break there a decrease in physical activity levels of schoolchildren in Florianópolis, where it was evidenced that this result due to climate issues during the intervention. A recent systematic review^37^ showed that interventions (increase in break time, availability of materials, and appointments on playgrounds markings) during breaks are associated with higher levels of physical activity. Therefore, that breaks during school hours can be an ideal time for interventions to promote physical activity. Not only do we need to consider the interventions, but also to consider the age group since with increasing age the effects of interventions on the interval decrease^35–37^. However, the literature showed inconsistent results on interventions for increasing physical activity and decreasing sedentary time among children^38,39^.

Active transport is an effective way for children to stay active^40^, but some barriers or facilitators can influence this domain^12^. Our study found associations between active transport and higher levels moderate-intensity, vigorous- intensity, and moderate-to-vigorous-intensity physical activity, which agrees with a study de Ferrari et al.^41^ with schoolchildren from São Paulo. The authors showed greater schoolchildren's achieving the physical activity recommendations when they had a built environment around the school conducive to active transportation. The same occurred with the study by Croocks et al.^23^ using objective measures of physical activity and found that there were 81% higher odds for girls to be more active when schools provide means for carrying out active transport. These results demonstrate an important role in encouragement and investments in active transport for meet with physical activity recommendations.

There were associations between access to the school’s physical facilities of with the increased in light-intensity and vigorous-intensity, as well as an increase in the odds of reaching the physical activity guideline, which agrees with the previous studies, in which other evidence^41–43^ showed that students were more likely to be more active when schools had greater numbers of facilities and better conditions of use. Such associations could be explained by the high variability in access between different school facilities, which would lead to increases in the different intensities of physical activity in different magnitudes^41^. The use of a high variety of facilities and dependencies can promote higher levels of physical activity for children, as well as investing in better structures are points where schools can intervene in a practical way.

From this perspective, policies for changes in school should be adopted and implemented. For instance, increased activities inside of school hours and varied activities, as well as increased time for physical activity breaks, structural changes in school facilities, and more equipment. In addition, structural changes in sidewalk improvements and traffic signals can promote more active transportation to school.

In our study, we had an objective method of measuring the physical activity of schoolchildren, but the present study has some limitations. First, this study has the cross-sectional design in which we cannot establish a cause-effect relationship. Second point is that our sample does isn’t representative and included schoolchildren from São Caetano do Sul, which has the largest human development index (HDI) of Brazil, therefore results cannot be generalizable to other populations. Third point is that the questionnaires of school environment answered by the principals and the sociodemographic questions answered by parents may suffer from memory bias. Fourth point is that the accelerometry data was downloaded at a sampling rate of 80 Hz that could overestimate physical activity in children^44^.

## Conclusions

We found in our study associations between environmental indicators school (policies and practices, extracurricular activities, and active break, and access to facilities) with increased levels of activity intensities physical. We can highlight the role of the school environment in promoting physical activity and health among schoolchildren. Policy promotion and implementation interventions public can help in a deeper understanding of this issue to provide data for the population and policy makers. In addition, the school environment may have an important role in allowing children, who spend part of their day in school, to have a more active lifestyle.

## Data Availability

Data are available upon reasonable request from the corresponding author.

## References

[1] Rodriguez-Ayllon M (2019). Role of physical activity and sedentary behavior in the mental health of preschoolers, children and adolescents: A systematic review and meta-analysis. Sports Med..

[2] Poitras VJ (2016). Systematic review of the relationships between objectively measured physical activity and health indicators in school-aged children and youth. Appl. Physiol. Nutr. Metab..

[3] Sampasa-Kanyinga H (2020). Combinations of physical activity, sedentary time, and sleep duration and their associations with depressive symptoms and other mental health problems in children and adolescents: A systematic review. Int. J. Behav. Nutr. Phys. Act..

[4] Andermo S (2020). School-related physical activity interventions and mental health among children: A systematic review and meta-analysis. Sports Med. Open.

[5] Guthold R, Stevens GA, Riley LM, Bull FC (2020). Global trends in insufficient physical activity among adolescents: A pooled analysis of 298 population-based surveys with 1.6 million participants. Lancet Child Adolesc. Health.

[6] Bauman AE, Sallis JF, Dzewaltowski DA, Owen N (2002). Toward a better understanding of the influences on physical activity: The role of determinants, correlates, causal variables, mediators, moderators, and confounders. Am. J. Prev. Med..

[7] Sallis JF (2006). An ecological approach to creating active living communities. Annu. Rev. Public Health.

[8] Morton KL, Atkin AJ, Corder K, Suhrcke M, van Sluijs EM (2016). The school environment and adolescent physical activity and sedentary behaviour: A mixed-studies systematic review. Obes. Rev..

[9] Langford R (2015). The world health organization's health promoting schools framework: A cochrane systematic review and meta-analysis. BMC Public Health.

[10] Morton KL (2016). School polices, programmes and facilities, and objectively measured sedentary time, LPA and MVPA: Associations in secondary school and over the transition from primary to secondary school. Int. J. Behav. Nutr. Phys. Act..

[11] van Sluijs EM (2011). School-level correlates of physical activity intensity in 10-year-old children. Int. J. Pediatr. Obes..

[12] Ferrari GL (2016). Correlates of moderate-to-vigorous physical activity in Brazilian children. J. Phys. Act. Health.

[13] Katzmarzyk PT (2013). The international study of childhood obesity, lifestyle and the environment (ISCOLE): Design and methods. BMC Public Health.

[14] Ferrari GLM, Matsudo V, Katzmarzyk PT, Fisberg M (2017). Prevalence and factors associated with body mass index in children aged 9–11 years. J. Pediatr. (Rio J.).

[15] Ferrari GL (2015). Moderate-to-vigorous physical activity and sedentary behavior: Independent Associations with body composition variables in Brazilian children. Pediatr. Exerc. Sci..

[16] Colley R, Connor Gorber S, Tremblay MS (2010). Quality control and data reduction procedures for accelerometry-derived measures of physical activity. Health Rep..

[17] Trost SG, Loprinzi PD, Moore R, Pfeiffer KA (2011). Comparison of accelerometer cut points for predicting activity intensity in youth. Med. Sci. Sports Exerc..

[18] Evenson KR, Catellier DJ, Gill K, Ondrak KS, McMurray RG (2008). Calibration of two objective measures of physical activity for children. J. Sports Sci..

[19] Barreira TV (2015). Identifying children's nocturnal sleep using 24-h waist accelerometry. Med. Sci. Sports Exerc..

[20] Tudor-Locke C, Barreira TV, Schuna JM, Mire EF, Katzmarzyk PT (2014). Fully automated waist-worn accelerometer algorithm for detecting children's sleep-period time separate from 24-h physical activity or sedentary behaviors. Appl. Physiol. Nutr. Metab..

[21] Bull FC (2020). World health organization 2020 guidelines on physical activity and sedentary behaviour. Br. J. Sports Med..

[22] Tudor-Locke C (2015). Improving wear time compliance with a 24-hour waist-worn accelerometer protocol in the international study of childhood obesity, lifestyle and the environment (ISCOLE). Int. J. Behav. Nutr. Phys. Act..

[23] Crooks N (2021). Association between the school physical activity environment, measured and self-reported student physical activity and active transport behaviours in Victoria, Australia. Int. J. Behav. Nutr. Phys. Act..

[24] Gomes TN (2014). Correlates of sedentary time in children: A multilevel modelling approach. BMC Public Health.

[25] Joint Consortium for School Health: Healthy School Planner. 2012. Available at: www.healthyschoolplanner.uwaterloo.ca.

[26] Cameron R (2007). Integrating public health policy, practice, evaluation, surveillance, and research: The school health action planning and evaluation system. Am. J. Public Health.

[27] Silva LJ (2016). The prevalence of physical activity and its associated effects among students in the Sao Paulo public school network, Brazil. Cien Saude Colet.

[28] Amornsriwatanakul A, Lester L, Rosenberg M, Bull F (2021). School policies and practices associated with Thai children's overall and domain specific physical activity. PLoS One.

[29] Batista M, Mondini L, Jaime PC (2017). Actions of the school health program and school meals in the prevention of childhood overweight: Experience in the municipality of Itapevi, Sao Paulo State, Brazil, 2014. Epidemiol. Serv. Saude.

[30] Matsudo SM (2003). The Agita Sao Paulo program as a model for using physical activity to promote health. Rev. Panam. Salud Publica..

[31] de Santiago LM, Rodrigues MT, de Oliveira Junior AD, Moreira TM (2012). School health program implementation in fortaleza-CE: Performance of the family health strategy staff. Rev. Bras. Enferm..

[32] Bourke M, Hilland TA, Craike M (2020). Factors associated with the institutionalization of a physical activity program in Australian elementary schools. Trans. Behav. Med..

[33] O'Hara Tompkins N (2020). Translating school physical education and activity policies into practice: A case study. Transl. J. Am. Coll. Sports Med..

[34] Parrish AM, Chong KH, Moriarty AL, Batterham M, Ridgers ND (2020). Interventions to change school recess activity levels in children and adolescents: A systematic review and meta-analysis. Sports Med..

[35] Erwin HE, Ickes M, Ahn S, Fedewa A (2014). Impact of recess interventions on children's physical activity: A meta-analysis. Am. J. Health Promot..

[36] Costa B (2019). The effect of an intervention on physical activity of moderate-and-vigorous intensity, and sedentary behavior during adolescents' time at school. Rev. Bras. Epidemiol..

[37] Suga ACM, Silva A, Brey JR, Guerra PH, Rodriguez-Anez CR (2021). Effects of interventions for promoting physical activity during recess in elementary schools: A systematic review. J. Pediatr. (Rio. J.).

[38] Neil-Sztramko SE, Caldwell H, Dobbins M (2021). School-based physical activity programs for promoting physical activity and fitness in children and adolescents aged 6 to 18. Cochrane Database Syst. Rev..

[39] Rodrigo-Sanjoaquin J (2022). Effectiveness of school-based interventions targeting physical activity and sedentary time among children: A systematic review and meta-analysis of accelerometer-assessed controlled trials. Public Health.

[40] Ferrari G, Rezende LFM, Wagner GA, Florindo AA, Peres MFT (2020). Physical activity patterns in a representative sample of adolescents from the largest city in Latin America: A cross-sectional study in Sao Paulo. BMJ Open.

[41] Ferrari G, Rezende LFM, Florindo AA, Mielke GI, Peres MFT (2021). School environment and physical activity in adolescents from Sao Paulo city. Sci. Rep..

[42] de Rezende LF (2015). The role of school environment in physical activity among Brazilian adolescents. PLoS One.

[43] Haug E, Torsheim T, Sallis JF, Samdal O (2010). The characteristics of the outdoor school environment associated with physical activity. Health Educ. Res..

[44] Clevenger KA (2022). Impact of ActiGraph sampling rate on free-living physical activity measurement in youth. Physiol. Meas..

